# Correlation of the severity of coronary artery disease with patients' metabolic profile- rationale, design and baseline patient characteristics of the CorLipid trial

**DOI:** 10.1186/s12872-021-01865-2

**Published:** 2021-02-08

**Authors:** Efstratios Karagiannidis, Georgios Sofidis, Andreas S. Papazoglou, Olga Deda, Eleftherios Panteris, Dimitrios V. Moysidis, Nikolaos Stalikas, Anastasios Kartas, Anastasios Papadopoulos, Leandros Stefanopoulos, Haralambos Karvounis, Helen Gika, Georgios Theodoridis, Georgios Sianos

**Affiliations:** 1 First Department of Cardiology, AHEPA University Hospital, Aristotle University of Thessaloniki, St. Kiriakidi 1, 54636 Thessaloniki, Greece; 2 Laboratory of Forensic Medicine and Toxicology, School of Medicine, Aristotle University of Thessaloniki, 54124 Thessaloniki, Greece; 3 Biomic_AUTh, CIRI-AUTH Center for Interdisciplinary Research and Innovation Aristotle University of Thessaloniki, 57001 Thessaloniki, Greece; 4 Laboratory of Analytical Chemistry, Department of Chemistry, Aristotle University of Thessaloniki, Thessaloniki, Greece; 5 1St Propaedeutic Internal Medicine Department, AHEPA Hospital Aristotle University of Thessaloniki, Thessaloniki, Greece; 6 Lab of Computing, Medical Informatics and Biomedical Imaging Technologies, Aristotle University of Thessaloniki, 54124 Thessaloniki, Greece

**Keywords:** Metabolomics, Metabolic profile, Ceramides, Carnitines, Biomarkers, Coronary artery disease, SYNTAX score, Atherosclerosis, Acute coronary syndrome, PCI

## Abstract

**Background:**

Coronary artery disease (CAD) remains one of the leading causes of mortality and morbidity worldwide. As oxygen and nutrient supply to the myocardium significantly decrease during ischemic periods, important changes occur regarding myocardial intermediary energy metabolism. Metabolomics is an emerging field in systems biology, which quantifies metabolic changes in response to disease progression. This study aims to evaluate the diagnostic utility of plasma metabolomics-based biomarkers for determining the complexity and severity of CAD, as it is assessed via the SYNTAX score.

**Methods:**

Corlipid is a prospective, non-interventional cohort trial empowered to enroll 1065 patients with no previous coronary intervention history, who undergo coronary angiography in University Hospital AHEPA, Thessaloniki. Venous blood samples are collected before coronary angiography. State-of the-art analytical methods are performed to calculate the serum levels of novel biomarkers: ceramides, acyl-carnitines, fatty acids, and proteins such as galectin-3, adiponectin, and the ratio of apolipoprotein B/apolipoprotein A1. Furthermore, all patients will be categorized based on the indication for coronary angiography (acute coronary syndrome, chronic coronary syndrome, preoperative coronary angiography) and on the severity of CAD using the SYNTAX score. Follow-up of 12 months after enrollment will be performed to record the occurrence of major adverse cardiovascular events. A risk prediction algorithm will be developed by combining clinical characteristics with established and novel biomarkers to identify patients at high risk for complex CAD based on their metabolite signatures. The first patient was enrolled in July 2019 and completion of enrollment is expected until May 2021.

**Discussion:**

CorLipid is an ongoing trial aiming to investigate the correlation between metabolic profile and complexity of coronary artery disease in a cohort of patients undergoing coronary angiography with the potential to suggest a decision-making tool with high discriminative power for patients with CAD. To our knowledge, Corlipid is the first study aspiring to create an integrative metabolomic biomarkers-based algorithm by combining metabolites from multiple classes, involved in a wide range of pathways with well-established biochemical markers.

*Trial registration* CorLipid trial registration: ClinicalTrials.gov number: NCT04580173. Registered 8 October 2020—Retrospectively registered, https://clinicaltrials.gov/ct2/show/NCT04580173.

## Background

Atherosclerosis is a pathological process strongly correlated with metabolic disorders of lipid oxidation stress [1]. Its consequent inflammatory alterations on the endothelium could lead to coronary artery disease (CAD), including stable and acute coronary syndrome. Risk factors associated with the initiation and the propagation of the atherosclerotic process have been identified as a result of the intense investigation over the last decades in this scientific field. Apart from traditional risk factors, such as age, sex, race, smoking, LDL and HDL cholesterol, systolic blood pressure, family history of premature myocardial infarction, diabetes mellitus and triglycerides, there are multiple studies integrating novel biomarkers, such as a genetic ones, cardiac troponin-T, high sensitivity C-reactive protein, apolipoprotein B/ apolipoprotein A-1 ratio, homocysteine or coronary artery calcium in CAD risk stratification [2, 3].

However, despite the remarkable progress in diagnosing and treating CAD during the last decade, it remains the leading cause of morbidity and mortality worldwide [4], while prognostic prediction remains challenging in this cohort of patients. This could be attributed to a lack of effective diagnostic methods for CAD in its early stages, and a poor understanding of its pathophysiology. Thus, further studies are still warranted to increase our understanding of the complex pathophysiological mechanisms underlying CAD and to shed light on novel CAD risk parameters.

Recently emerging scientific evidence supports that cardiac metabolism disturbances indicate high probability of cardiovascular disease [5]. Hence, metabolomics, one of the newest and most rapidly evolving omics techniques, could be employed as a targeted tool for determining cardiac metabolism perturbation [6, 7]. According to the American Heart Association, cardiovascular metabolomics is a promising field able to provide detailed metabolic profiling of biospecimens and investigate both the intrinsic and extrinsic factors that contribute to CAD risk [8]. More specifically, multiple studies revealed that various metabolites such as BCAAs (branched-chain amino acids), specific unsaturated lipid species [8], trimethylamine-N-oxide [9], choline [10, 11], cysteine, N-acetylalanine and theophylline [12] are linked with an advanced cardiometabolic risk profile derived through metabolomics-based analysis using Liquid Chromatography-Mass Spectrometry or Nuclear Magnetic Resonance Spectroscopy. Furthermore, there are also data suggesting that other already established biochemical markers, such as cholesterol and triglycerides, have been strongly associated with CAD pathogenesis [6]. Therefore, their clinical importance in prediction and risk stratification of cardiovascular events should be thoroughly evaluated.

The rationale of this real-world observational study is by measuring metabolic profiles to expand our knowledge on the biochemical process of atherogenesis and to identify clinically significant metabolic biomarkers associated with the pathogenesis and the prognosis of CAD. Previous studies have already been conducted trying to establish potential correlations between metabolic profiling and CAD [11–14]. However, to the best of our knowledge, this is the first study aiming to associate the severity and the complexity of CAD, as assessed by the SYNTAX score [15] with patients’ metabolic profile. This study's ultimate goal is to suggest a clinical tool of personalized medicine based on the patients' metabolic fingerprint to distinguish the patients at high risk for adverse cardiovascular events.

## Methods

### Study design and population

CORLIPID (ClinicalTrials.gov Identifier: NCT04580173) is an investigator-initiated, prospective, non-interventional cohort trial involving patients undergoing coronary angiography.

Every trial procedure was conducted following the principles set by the declaration of Helsinki and the International Conference on Harmonization Guidelines for Good Clinical Practice [16]. The Scientific Committee of AHEPA University Hospital has approved the study protocol (reference number 12/13-06-2019). Every patient attested informed consent before being enrolled in our study.

A total of 1065 patients presenting to AHEPA University Hospital, who undergo scheduled or emergency coronary angiography, will be enrolled in the study. Patients with a history of previous Coronary Artery Bypass Graft or Percutaneous Coronary Intervention are excluded, as it is not possible to calculate the SYNTAX score for these patients. Detailed eligibility criteria are described in Table 1.

**Table 1:** Selection criteria for the enrollment in the CorLipid study

Inclusion criteria	Exclusion criteria
Patients undergoing coronary angiography without medical history of coronary artery disease Age > 18 years old Informed consent for study participation	Medical history of prior percutaneous coronary intervention Medical history of prior coronary artery bypass grafting Cardiopulmonary arrest Severe concomitant disease with a life expectancy less than 1 year

The following clinical characteristics were recorded for each patient: demographics, medical history and medication, as well as serum biochemical markers obtained on the enrollment day. Furthermore, patients were categorized into: (1) patients without CAD symptoms (undergoing preoperative coronary angiography prior to cardiac valve surgery), (2) patients with stable angina, (3) patients with acute coronary syndrome (unstable angina, non-ST elevation myocardial infarction (NSTEMI) and ST-elevation myocardial infarction (STEMI)).

In addition, coronary angiographic images of every patient were evaluated by two experienced independent interventional cardiologists blinded to the clinical aspects and the outcome of the patients and the SYNTAX score was calculated. Based on SYNTAX score, patients were classified into: (1) patients with low SYNTAX score (0–22), (2) patients with intermediate SYNTAX score (23–32) and (3) patients with high SYNTAX score (> 32) [15].

Venous blood sample was collected from all participants prior to coronary angiography. The blood collection vials were placed for incubation for 30 min. Immediately after centrifugation at 4000 rpm for 10 min at room temperature, the serum samples were stored at − 80 °C.

Telephone follow-up will be performed on the 12^th^ month after study entrance for each patient to document the occurrence of CAD symptoms, major adverse cardiovascular and cerebrovascular events (MACCE-cardiovascular death, acute myocardial infarction, need for revascularization, stroke episode) or bleeding complications.

### Biochemical analysis-metabolomics

Metabolomics-based analysis using Gas Chromatography tandem Mass Spectrometry (GC–MS) as well as targeted methods based on Liquid Chromatography tandem Mass Spectrometry (LC–MS) and Gas Chromatography with Flame Ionization Detector (GC-FID) will be performed. The untargeted approach will be used as it offers the advantages of novel biomarkers discovery from a wide range of compounds. The quantification of four ceramide species (C18:0/16:0, C18:0/18:0, C18:0/24:0 and C18:0/24:1) with their respective ratios and of fifteen short-, medium- and long chain acylcarnitines in serum CAD patients will be achieved with targeted methods by applying a developed RPLC and HILIC LC –MS/MS method, respectively. The composition of total fatty acids will be quantitatively measured in patients’ serum samples using a developed GC-FID based method. In addition, protein markers associated with CAD, namely Galectin-3, adiponectin, NGAL and ApoB/ ApoΑ-Ι will be measured by enzyme-linked immunosorbent assay (ELISA) in serum patients’ samples in order to enhance the predictive value of the CorLipid panel.

### Statistical considerations

#### Sample size estimation

Sample size estimation for studies utilizing metabolomic data in various degrees is not straightforward as effect sizes are not readily established in this very active area of research. The primary endpoint of the study is to discover potential correlations of clinical types of Coronary Artery Disease with patients' metabolic profile. Syntax score as a descriptor of CAD complexity could be probably correlated with an array of known and novel small molecule biomarkers to determine if the varying level of CAD complexity is related to metabolism.

Targeted analyses on concentrations of known molecules, such as Ceramides, Galectin-3, Adiponectin, NGal etc. will be the majority of the metabolic profile analyses conducted and we intent to explore potential correlations of them with the Syntax score. This multiparametric approach hinders a more accurate estimation of an effect size, as there is a multitude of factors at play. Thus, using a pilot study of 100 patients, we performed a linear multiple regression random model to establish an effect size for a direct effect on the SYNTAX score from various parameters. In this pilot study we chose 10 predictors (concentrations of current and potential biomarkers and categorical parameters) as in the expanded study we will aim to choose smaller more concise models with less than 10 predictors of better biological interpretations.

Using the G*Power tool [17] and a calculated effect based on a regression model R^2^ of 0.370, an effect size of H1 p^2^ 0.30 was yielded. We expect other factors, not explicitly measured by this study, to also independently influence CAD complexity as measured by Syntax score, so we arbitrary assume a Η0 ρ^2^ of 0.20. So for an A of 0.01 and a Power of 0.95 a sample size of 1014 is sufficient. This initial sample size will be increased by 5%, as some study participants might be lost to follow-up. Hence, we aim for a total of 1065 patients.

### Statistical analysis

Normally distributed data will be analyzed using parametrical tests, such as Student *t*-test and for non-normally distributed data nonparametric test such are Mann–Whitney and multiparametric Kruscal-Wallis H test will be utilized, as indicated by the Kolmogorov-Smirnoff Test for normality. Categorical variables will be compared using the χ^2^ test. Continuous data will be presented as mean ± SD, whereas categorical variables will be displayed as counts and percentages. Univariable, multivariable and correlations analysis will be carried out to clarify independent predictors of various CAD characteristics, biochemical measurements and associations with SYNTAX score, metabolic biomarkers individually as well as follow up MACCE.

Linear and logistic regression, using stratified bootstrapping if needed to account for potential non-parametric data, will be used to identify independent predictors of CAD characteristics and Syntax Score. Receiver operating characteristic (ROC) curve analysis -and derived area under the curve (AUC) -will be utilized when appropriate. Cox regression analyses and Kaplan–Meier survival will be calculated for the 1 year follow-up for Syntax score groups and to check the validity of potential predictive models that will emerge, with the difference between different patient sub-groups analyzed by the log-rank test. Patients that are lost to follow-up will be censored at their last study encounter. The level of statistical significance will be considered at a = 0.05 and every statistical test will be performed via SPSS v. 26.

#### Endpoints

The study primarily aims to combine clinical characteristics with established and novel biomarkers, in order to develop a risk prediction model, which could optimally identify patients at high risk for CAD and most importantly, patients at high risk for complex CAD based on their metabolite signatures.

**Fig. 1:**
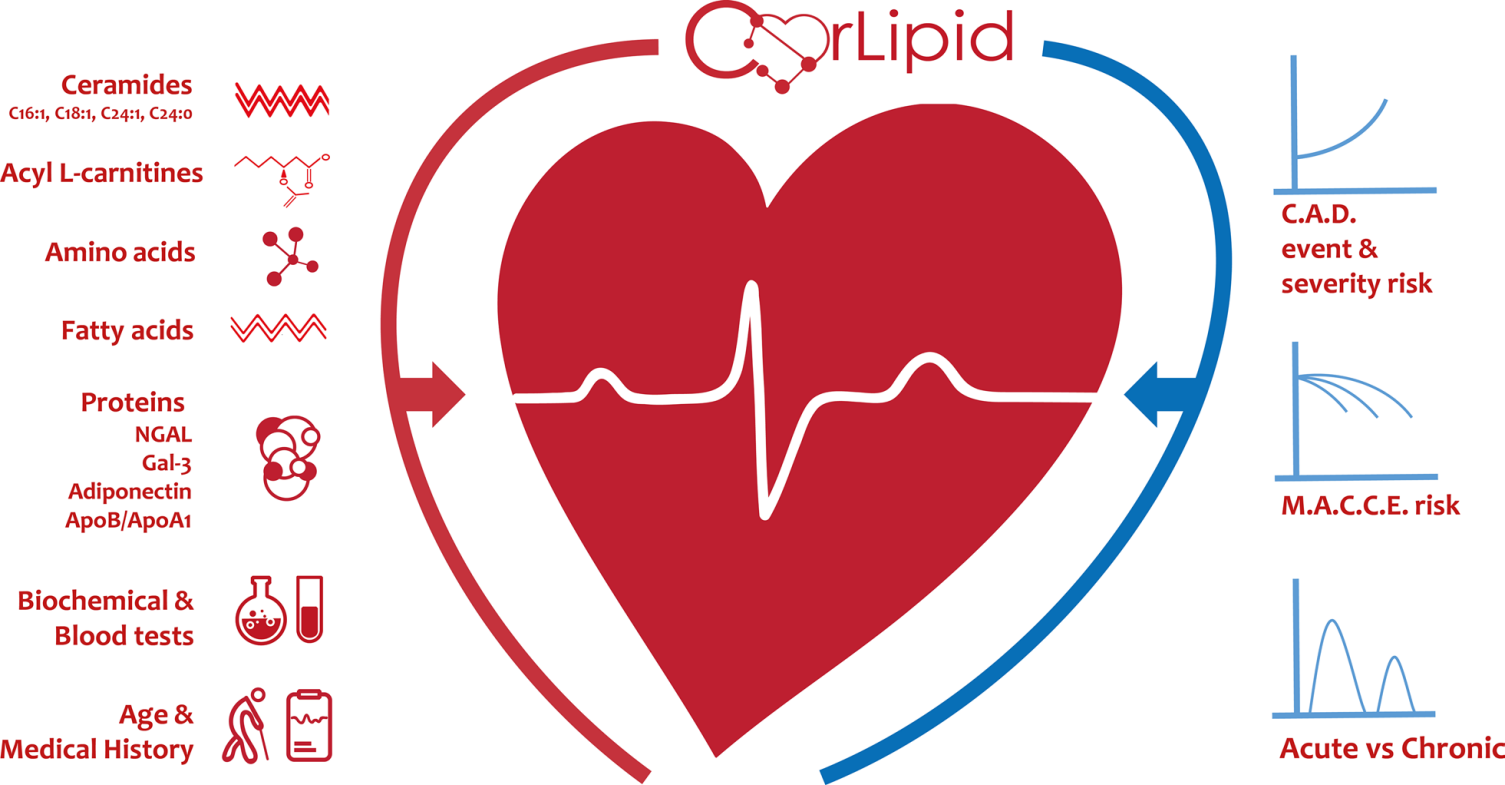
Visual overview of the CorLipid study. This figure is original and, therefore, permission for publication was not needed to be obtained from a third-party

A secondary endpoint of this study is the identification of a biomarkers’ panel for the distinction of acute from chronic coronary syndromes by detecting metabolite pattern differences. Finally, all patients will be followed-up via telephone for 12 months for any MACCE to develop a new prognostic metabolomics-based model for CAD patients (Fig. 1).

### Patient recruitment and baseline characteristics

From o till September 2020, a total of 596 patients were enrolled in the study. Mean age was 63.71 (SD ± 12.68) years. 266 patients (44.63%) presented with acute coronary syndrome, of whom 106 patients were diagnosed with STEMI, 99 patients were diagnosed with NSTEMI and 66 patients suffered from unstable angina. 330 patients presented with Chronic Coronary Syndromes. Hypertension was found in 56.5% of patients. About 28.7% were diabetics, while dyslipidemia was observed in 38.4%. Smoking was reported by 41.5% of patients. During the aforementioned recruitment period the mean SYNTAX score of the patients was 11.5. The baseline characteristics of study participants are shown in Table 2. Completion of enrollment is expected until May 2021.

**Table 2:** Baseline characteristics of study participants

Βaseline characteristics	Ν	%
Male gender	438	73.5
*Age groups*		
65 <	318	53.5
65 >	277	46.5
Hypertension	337	56.5
Diabetes mellitus	171	28.7
Dyslipidemia	229	38.4
Acute coronary syndrome	266	44.5
NSTEMI	99	16.6
STEMI	106	17.8
*Angina*		
Unstable	66	11.1
Stable	96	16.1
Use of statins	286	47.5
*SYNTAX score groups*		
0–22	490	82.2
23–32	72	12.1
> 32	34	5.7

## Discussion

CorLipid is an ongoing trial aspiring to create a unique tool to complement traditional risk factor assessment by applying novel metabolomics-based techniques. These techniques enable combined profiling of different metabolites that could serve as disease biomarkers with the potential to inform clinical decision-making for patients with CAD.

Currently it is an established concept that alterations in the serum metabolome may be detectable in at-risk individuals and other relative studies have already demonstrated strong correlation between metabolites’ levels and CAD risk [8]. For instance, a multi-center trial in China determined the plasma metabolomic profiles of 2,324 patients undergoing coronary angiography by liquid chromatography-quadrupole time-of-flight mass spectrometry and made 12 cross-comparisons to and within CAD identifying 89 differential metabolites closely related to the occurrence and development of CAD [14]. Distinct plasma ceramide ratios have been found to be significant predictors of cardiovascular death both in patients with stable CAD and acute coronary syndrome, over and above currently used lipid markers [18, 19].

Another study conducted by Jiang et al. [13] analyzed the serum metabolomics pertubation on atherosclerotic plaque stability of 252 patients sustaining acute coronary syndrome and also undergoing emergency coronary angiography and revealed significantly altered betaine, acetylcarnitine, 1-heptadecanoyl-sn-glycero-3-phosphocholine, and isoundecylic acid levels in patients with vulnerable plagues. Furthermore, they proposed a combined model of serum betaine and ejection fraction as a potential diagnostic biomarker to distinguish stable from vulnerable plagues patients. Eventually, an insight from the STABILITY trial [20] featured a Ceramide‐Phospholipid based risk (CERT2) score as a risk indicator in patients with stable coronary heart disease.

The present study seeks to contribute to the literature as mentioned above by examining the association between the complexity and the severity of CAD with novel metabolomics biomarkers in a large patient cohort with the potential to implement a sensitive metabolomics-based risk score for the assessment of such patients. State-of-the-art advanced metabolomics-based techniques, which enable targeted and untargeted profiling of a wide range of metabolites (ceramides, acyl-carnitines, fatty acids, small molecules and polar compounds) and protein markers serving as precursors and products in different metabolic pathways, will be employed aiming to reveal potential metabolites differences amongst the participants. Metabolomic profiling will not only provide useful insights into the understanding of the underlying mechanisms of CAD by uncovering novel disease-related pathways, but it will also enable us to accurately identify patients at risk of CAD and predict patients’ response to specific treatments. To this end, the metabolomics-based algorithm will be combined with patients’ clinical and angiographic characteristics in order to enhance and support clinical diagnosis and disease prognosis.

In this trial, there are also some limitations that should be acknowledged. The main limitation of our study is the single-center character of its design and thus covering specific geographical location. Our study population mainly consists of Greek patients. In future studies, the scope could be widened to include other ethnicities within Europe and other races such as Africans in order to account for inherent variability of different patient populations. Additionally we should consider that potential differences in the nutritional and medical history among the participants could make difficult the interpretation of our results, since they could affect their metabolism. In order to identify such confounders, detailed information about dietary habits and medical treatment will be recorded upon recruitment.

Future studies should include the integration of metabolomics platform with other "omics" techniques directing to the application of multi- “omics”. In that way the cardiovascular scientific community will gain deep insight into pathophysiological interactions of genes, proteins, metabolites and disease states, while promoting individualized medicine.

In conclusion, deciphering the details of serum metabolome has the potential to identify novel cardiovascular disease biomarkers, which can accurately predict the risk for coronary artery disease and subsequent cardiovascular events, and to significantly alter the risk stratification strategies of coronary artery disease.

## Data Availability

Data are available from Georgios Sianos (e-mail: gsianos@auth.gr) upon reasonable request and with permission of AHEPA University Hospital.

## Abbreviations

CAD: Coronary artery disease
STEMI: ST elevation myocardial infarction
NSTEMI: Non-ST elevation myocardial infarction
MACCE: Major adverse cardiovascular and cerebrovascular events
